# Thermotolerance effect of plant growth-promoting *Bacillus cereus* SA1 on soybean during heat stress

**DOI:** 10.1186/s12866-020-01822-7

**Published:** 2020-06-22

**Authors:** Muhammad Aaqil Khan, Sajjad Asaf, Abdul Latif Khan, Rahmatullah Jan, Sang-Mo Kang, Kyung-Min Kim, In-Jung Lee

**Affiliations:** 1 School of Applied Biosciences, Kyungpook National University, Daegu, 41566 Republic of Korea; 2 Natural and Medical Sciences Research Center, University of Nizwa, 616 Nizwa, Oman

**Keywords:** *B. cereus* SA1, Heat stress, Phytohormone, Amino acid, HSP expression, Soybean

## Abstract

**Background:**

Incidences of heat stress due to the changing global climate can negatively affect the growth and yield of temperature-sensitive crops such as soybean variety, Pungsannamul. Increased temperatures decrease crop productivity by affecting biochemical, physiological, molecular, and morphological factors either individually or in combination with other abiotic stresses. The application of plant growth-promoting endophytic bacteria (PGPEB) offers an ecofriendly approach for improving agriculture crop production and counteracting the negative effects of heat stress.

**Results:**

We isolated, screened and identified thermotolerant *B. cereus* SA1 as a bacterium that could produce biologically active metabolites, such as gibberellin, indole-3-acetic acid, and organic acids. SA1 inoculation improved the biomass, chlorophyll content, and chlorophyll fluorescence of soybean plants under normal and heat stress conditions for 5 and 10 days. Heat stress increased abscisic acid (ABA) and reduced salicylic acid (SA); however, SA1 inoculation markedly reduced ABA and increased SA. Antioxidant analysis results showed that SA1 increased the ascorbic acid peroxidase, superoxide dismutase, and glutathione contents in soybean plants. In addition, heat stress markedly decreased amino acid contents; however, they were increased with SA1 inoculation. Heat stress for 5 days increased heat shock protein (HSP) expression, and a decrease in *GmHSP* expression was observed after 10 days; however, SA1 inoculation augmented the heat stress response and increased HSP expression. The stress-responsive *GmLAX3* and *GmAKT2* were overexpressed in SA1-inoculated plants and may be associated with decreased reactive oxygen species generation, altered auxin and ABA stimuli, and enhanced potassium gradients, which are critical in plants under heat stress.

**Conclusion:**

The current findings suggest that *B. cereus* SA1 could be used as a thermotolerant bacterium for the mitigation of heat stress damage in soybean plants and could be commercialized as a biofertilizer only in case found non-pathogenic.

## Background

Changes in the climate with increases in the temperature are major problems in many parts of the world [1]. According to the Intergovernmental Panel on Climate Change (IPCC, 2007) report, an estimated increase of 0.20 °C/year for the next two decades will be detrimental to agricultural growth and yield [2]. Similarly, an increase in the temperature from 3 to 4 °C can cause a decrease in agricultural crop productivity up to 15–35% in Asia and Africa and 25–35% in the Middle East [3]. Therefore, the current trends in global warming, which can affect various ecosystems and pose a considerable threat to future food security and staple food production.

Increased temperature or heat stress affects various characteristics of agricultural crop plants, including germination and fruiting patterns [4, 5]. Heat stress causes biochemical, physiological, molecular, and morphological changes that can adversely affect plant growth, biomass, productivity, and yield either individually or in combination with other abiotic stresses [4–6]. Biochemical changes include changes in the fluidity of membranes, organizational changes in the cellular structure, structural changes in amino acids, an increase or decrease in the concentration of metabolites and osmolytes, an increase in stress hormones such as abscisic acid (ABA), a decrease in defense hormones such as salicylic acid (SA), and the production of harmful reactive oxygen species (ROS) and antioxidants [7]. ROS generation leads to membrane damage (lipid peroxidase), triggering stress responses in the defensive antioxidant system and inducing oxidative damage through glutathione (GSH), ascorbic acid peroxidase (APX), and superoxide production [8, 9]. Molecular changes include the alteration of genes involved in the protection against heat stress. These genes are responsible for the expression of osmoprotectants, detoxifying enzymes, and transporters and upregulate the expression of heat shock proteins (HSPs), stress-induced proteins, or stress proteins, playing a key role in conferring stress tolerance when the plants are exposed to stresses [10–12]. Similarly, the auxin influx carrier LAX3 (*GmLAX3*) and potassium channel AKT2 (*GmAKT2*) genes also enhance antioxidant defense, increase photosynthesis in plants, and protect plants under various environmental stress conditions such as heat stress [13].

Heat stress tolerance may be improved through genetic engineering, breeding programs, tissue culture methods, and chemical fertilizer applications, which are time consuming and costly and have adverse effects on the environment [12, 14–16]. The use of plant growth-promoting endophytic bacteria (PGPEB) is an alternative and ecofriendly approach for improving agriculture crop production by ameliorating the negative effect of high temperatures on economically important plant species worldwide [17–20]. Previously, a number of researchers have reported the use of plant growth-promoting endophytic bacteria to enhance tolerance to heat stress in plants such as sorghum [19], chickpea [21], wheat [20, 22], tomato [23], and potato [24]. Furthermore, PGPEB can synthesize phytohormones that help increase tolerance against heat stress by enhancing biofilm formation, reducing ABA levels, and increasing HSP levels [17, 19–22, 25].

Globally, soybean is an important commercial crop, and 120.48 million hectares of land are used for soybean cultivation throughout the world [26]. Nutritionally, soybean contains vitamins, minerals, fibers, and antioxidant compounds [27]. Soybean were used in various products like tofu, soy sauce, bean paste, soybean oil and soy milk [28]. Lipid and protein are the major components of soybean and about 92–100% of soybean protein is easily digest by human [29]. In current study soybean verity Pungsannamul, the most favored sprout cultivar in Korea contain 348 mg amino acid per gram, 2556 μg/g of isoflavone, and rich source of ß-carotene, lutein and vitamins [30]. Soybean is sensitive to high temperatures, which can cause changes in metabolomics [31] and antioxidant production [32]. Heat stress adversely affects soybean growth, photosynthesis, and productivity, especially in tropical and semi-arid tropical regions [17, 33]. A 1% increase in the average growing season temperature has been reported to lead to a 3.1% decrease in soybean yield [34]. Several studies such as those of Srivastava et al. [21], Park et al. [17], Abd El-Daim et al. [22], and Ali et al. [19, 20] have emphasized the use of beneficial microorganisms, which can tolerate heat stress and augment the growth and productivity of crop plants. However, further studies are needed to elucidate the effect of PGPEB on the growth attributes, chlorophyll contents, and HSPs of soybean plants under heat stress. Therefore, the present study aimed to (i) isolate and identify plant growth-promoting and thermotolerant bacteria that can mitigate heat stress and enhance plant growth and (ii) analyze antioxidant components such as APX, superoxide dismutase (SOD), lipid peroxidation (LPO), reduced GSH, and phytohormones (ABA and SA) and the transcription of responsive genes (*GmHSP*, *GmLAX3*, and *GmAKT2*) in soybean with or without inoculation of bacterium during heat stress.

## Results

### Isolation of competent bacteria, screening for IAA, phosphate solubilization, and siderophore production, and bioassay assessment

From the roots of *O. biennis*, *C. ficifolium*, *A. princeps*, *and E. crus-galli*, 59 endophytic isolates were isolated (Supplementary Table 1). These isolates were screened for different plant growth-promoting traits, i.e., IAA, GA, and siderophore production and phosphate solubilization. However, only 13 isolates showed multiple plant growth-promoting traits (Supplementary Table 1). Based on their plant growth-promoting traits, thirteen isolates were further applied to *Waito-C* rice for measuring their germination/seedling potential. A total of 8 isolates were found to increase growth attributes significantly.

### Screening for thermotolerant bacteria

**Fig. 1:**
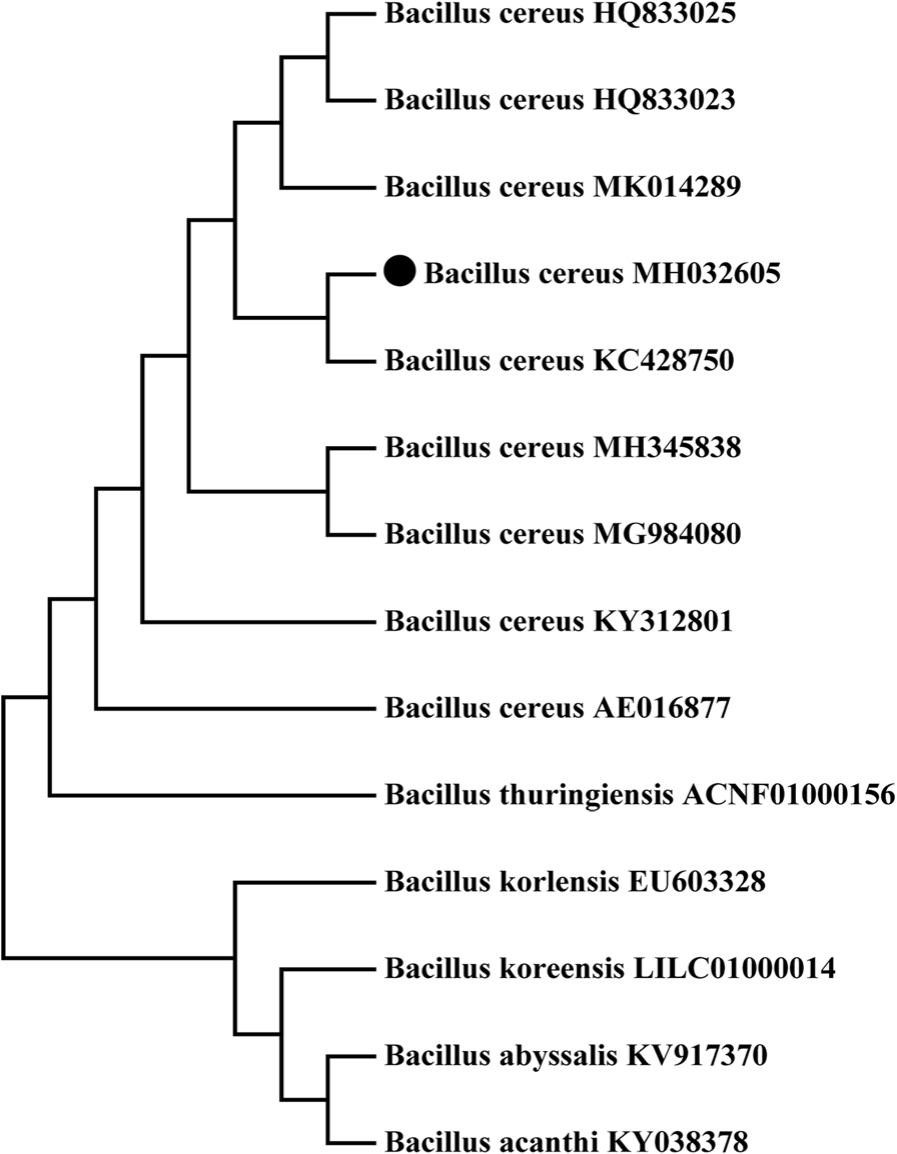
Phylogenetic tree based on 16S rRNA sequences. A phylogenetic tree based on the 16S rRNA sequences of the endophytic bacterial strain SA1 isolated from the roots of *Echinochloa crus-galli* (L.) Beauv was constructed

**Fig. 2:**
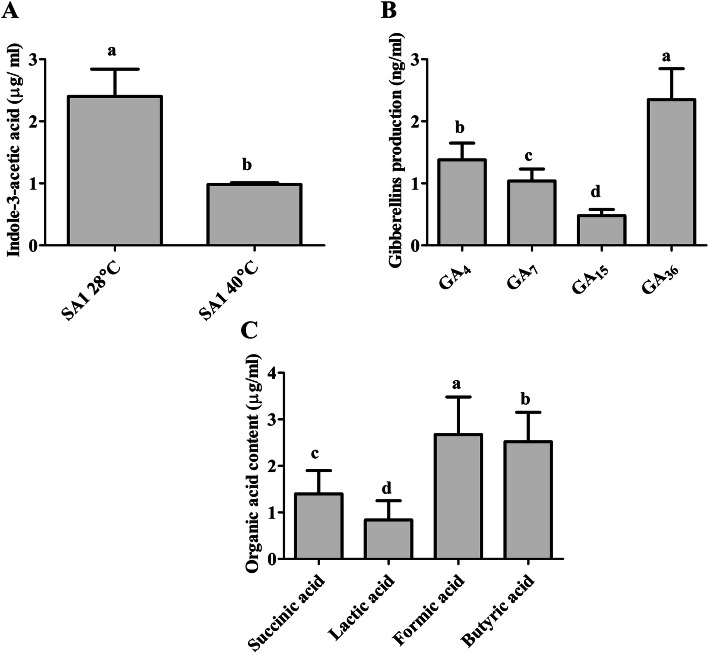
Quantification of indole-3-acetic acid (IAA), gibberellins (GAs) and organic acids produced by the endophytic bacterial strain SA1. **a** Indole-3-acetic acid, **b** Gibberellins, **c** Organic acid. Each data point is the mean of three replicates. Error bars represent standard errors. The bars with different letters are significantly different from each other as evaluated by DMRT analysis

All the selected isolates were examined for their ability to grow at 25 °C, 30 °C, 35 °C, 40 °C, and 45 °C on solid and in liquid LB media. The results showed that the growth of the isolates was normal at 35 °C on solid and in liquid media; however, increasing the temperature to 40 °C hampered the growth of all isolates. Furthermore, an increase in the temperature to 45 °C reduced the growth rate of all isolates, and only SA1 (isolated from the root of *E. crus-galli*) showed tolerance against heat stress (S. Fig 1 and S. Fig. 2). Therefore, isolate SA1 was selected for further investigation. For molecular identification and phylogenetic analysis of strain SA1, the 16S rRNA gene was amplified, sequenced, and compared with the database of known 16S rRNA gene sequences and BLAST search tool of NCBI data base/EzTaxon was used to determine the nucleotide sequence homology of the targeted bacterial isolate. Our results revealed that SA1 exhibited a high 16S rRNA gene sequence identity (99%) to *Bacillus cereus*. The SA1 16S rRNA gene sequence was submitted to NCBI with the GenBank accession no. MH032605 (Fig. 1).

### In vitro IAA, GA, and organic acid production from bacterial isolates and GC/MS-SIM analysis

Phytohormones (IAA and GAs) in the culture filtrate (CF) of isolate SA1 was quantified by GC/MS, and organic acids were quantified by HPLC. The selected isolate SA1 produced significant amounts of IAA (2.4 ± 0.4 μg/ml) and GA [bioactive: GA4 (1.3 ± 0.2 μg/ml) and GA7 (1.04 ± 0.1 μg/ml); non-bioactive: GA15 (0.48 ± 0.4 μg/ml) and GA36 (2.35 ± 0.5 μg/ml)] (Fig. 2a&b). Organic acid analysis revealed that the CF of SA1 contained lactic acid (0.84 ± 0.4 μg/ml), butyric acid (2.5 ± 0.6 μg/ml), formic acid (2.6 ± 0.8 μg/ml), and succinic acid (1.4 ± 0.5 μg/ml) (Fig. 2c).

### Plant growth conditions

**Fig. 3:**
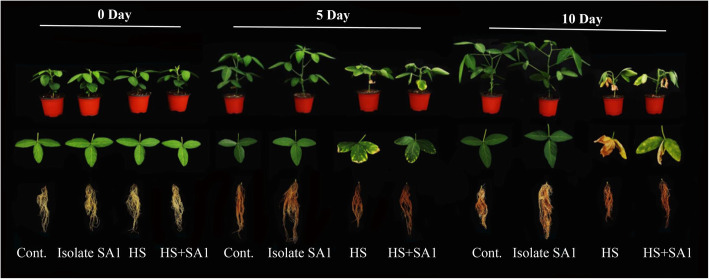
Effect of the bacterial strain SA1 on soybean plant growth. The effects of the selected endophytic bacterial isolate (SA1) on the growth of soybean plants with and without SA1 inoculation under heat stress were determined at different time points (0, 5, and 10 days)

In comparison with the control, bacterial inoculation significantly enhanced the thermotolerance of soybean plants as demonstrated by changes in the leaf ultrastructure, and soybean biomass (fresh/dry weight, root/shoot length) after 5 and 10 days of heat stress (Fig. 3; Table 1). Under heat stress, a decrease in the shoot length (40.55%), root length (14.53%), fresh and dry weight (38 and 24%, respectively) were observed. Similarly, SA1-inoculated plants showed significant increase in shoot length (15.08%), root length (14.63%), fresh and dry weight (27.28 and 12.39%, respectively) after 5 days under normal conditions as compared to control in control as well as heat treated plants (Table 1) A similar pattern was observed after 10 days of soybean growth with SA1 inoculation under heat stress (Table 1; Fig. 3). A significant difference were observed in the root, shoot length and fresh/dry biomass, of inoculated and non-inoculated soybean plants at 10 days under normal and stress conditions.

**Table 1:** Demonstrate the effect of heat stress on shoot length (SL), root length (RL), fresh weight (FW) and dry weight (DW) in soybean plants with and without inoculation of SA1 at different time point (0, 5 and 10 Days. Each data point is the mean of three replicates. Error bars represent standard errors. The bars represented with different letters are significantly different from each other as evaluated by DMRT analysis

	Cont.	SA1	HS	HS + SA1
**0 Days**				
**SL (cm)**	4.92 ± 0.4^a^	4.85 ± 0.4^b^	5 ± 08.5^a^	4.92 ± 0.4^a^
**RL (cm)**	6.56 ± 0.5^b^	7.32 ± 0.5^ab^	7.06 ± 0.6^ab^	7.83 ± 0.7^a^
**FW (g)**	5.13 ± 0.4^b^	6.41 ± 0.6^a^	7.09 ± 0.6^a^	6.65 ± 0.3^a^
**DW (g)**	0.70 ± 0.09^c^	0.86 ± 0.09^b^	0.90 ± 0.07^a^	0.75 ± 0.06^b^
**5 Days**				
**SL (cm)**	22.02 ± 0.5^b^	25.93 ± 0.5^a^	13.09 ± 0.6^d^	15.08 ± 0.6^c^
**RL (cm)**	26.14 ± 1.2^ab^	27.72 ± 2.0^a^	22.34 ± 1.9^c^	25.61 ± 2.3^b^
**FW (g)**	10.81 ± 0.7^b^	13.17 ± 0.8^a^	6.67 ± 0.5^d^	8.49 ± 0.5^c^
**DW (g)**	1.61 ± 0.2^a^	1.83 ± 1.2^a^	1.21 ± 1.4^b^	1.36 ± 1.2^b^
**10 Days**				
**SL (cm)**	25.3 ± 2.2^b^	30.4 ± 2.8^a^	15.7 ± 1.7^d^	18.9 ± 1.4^c^
**RL (cm)**	29.3 ± 3.2^b^	33.4 ± 4.1^a^	19.1 ± 2.1^d^	23.2 ± 1.4^c^
**FW (g)**	18.2 ± 1.6^b^	21.3 ± 1.5^a^	6.5 ± 1.5^c^	7.0 ± 1.8^c^
**DW (g)**	2.64 ± 1.8^b^	3.1 ± 1.9^a^	1.39 ± 0.9^d^	1.65 ± 0.8^c^

Chlorophyll analysis results revealed heat stress significantly decreased *Chl a* (26%), *Chl b* (34%), and carotenoid (43%) after 5 days. However inoculation of thermotolerant SA1 mitigated heat stress and increased *Chl a* (11.20%), *Chl b* (11.90%), and carotenoid (12.12%) compared with heat stressed control plants. A similar pattern was observed after 10 days for SA1-inoculated soybean plants grown under heat stress (Table 2). Furthermore, chlorophyll fluorescence results revealed that heat stress significantly decreased chlorophyll fluorescence from 11 to 40% after 5 and 10 days of heat stress compared with control plants. Inoculation with SA1 markedly increased the chlorophyll fluorescence (6.94 to 25%) of soybean plants (Table 2).

**Table 2:** Demonstrate the effect of heat stress on different chlorophyll contents and chlorophyll fluorescence in soybean plants with and without inoculation of SA1 at different time point (0, 5 and 10 Days). (A) Chlorophyll fluorescence (Fv/Fm) (B) *Chl a* (C) *Chl b* and (D) carotenoid. Each data point is the mean of three replicates. Error bars represent standard errors. The bars represented with different letters are significantly different from each other as evaluated by DMRT analysis

	Fv/Fm	***Chl a***	***Chl b***	Carotenoid
**0 Days**				
**Cont.**	0.84 ± 0.07^ab^	17.83 ± 2.5^a^	23.39 ± 2.5^a^	2.84 ± 0.6^a^
**SA1**	0.93 ± 0.04^a^	19.79 ± 3.7^a^	24.18 ± 3.0^a^	2.72 ± 0.4^a^
**HS**	0.8 ± 0.06^b^	18.37 ± 2.7^a^	23.79 ± 4.1^a^	2.99 ± 0.5^a^
**HS + SA1**	0.81 ± 0.02^ab^	18.98 ± 3.3^a^	24.39 ± 3.2^a^	2.86 ± 0.5^a^
**5 Days**				
**Cont.**	0.81 ± 0.08^b^	19.38 ± 1.4^a^	24.87 ± 4.2^a^	2.93 ± 0.6^a^
**SA1**	0.83 ± 0.06^a^	19.97 ± 1.6^a^	25.01 ± 3.3^a^	3.14 ± 0.4^a^
**HS**	0.72 ± 0.07^d^	14.28 ± 0.9^c^	16.38 ± 2.5^c^	1.65 ± 0.3^b^
**HS + SA1**	0.77 ± 0.06^c^	15.88 ± 0.8^b^	18.33 ± 4.4^b^	1.85 ± 0.3^b^
**10 Days**				
**Cont.**	0.81 ± 0.90^b^	20.59 ± 3.1^a^	25.61 ± 4.9^a^	3.32 ± 0.7^b^
**SA1**	0.84 ± 0.98^a^	21.99 ± 2.6^a^	26.01 ± 5.2^a^	3.91 ± 0.6^a^
**HS**	0.48 ± 0.04^d^	6.86 ± 1.4^c^	14.21 ± 2.1^b^	0.94 ± 0.5^d^
**HS + SA1**	0.6 ± 0.03^c^	9.25 ± 1.1^b^	15.28 ± 3.0^b^	1.26 ± 0.2^c^

### Quantification of plant endogenous phytohormones

**Fig. 4:**
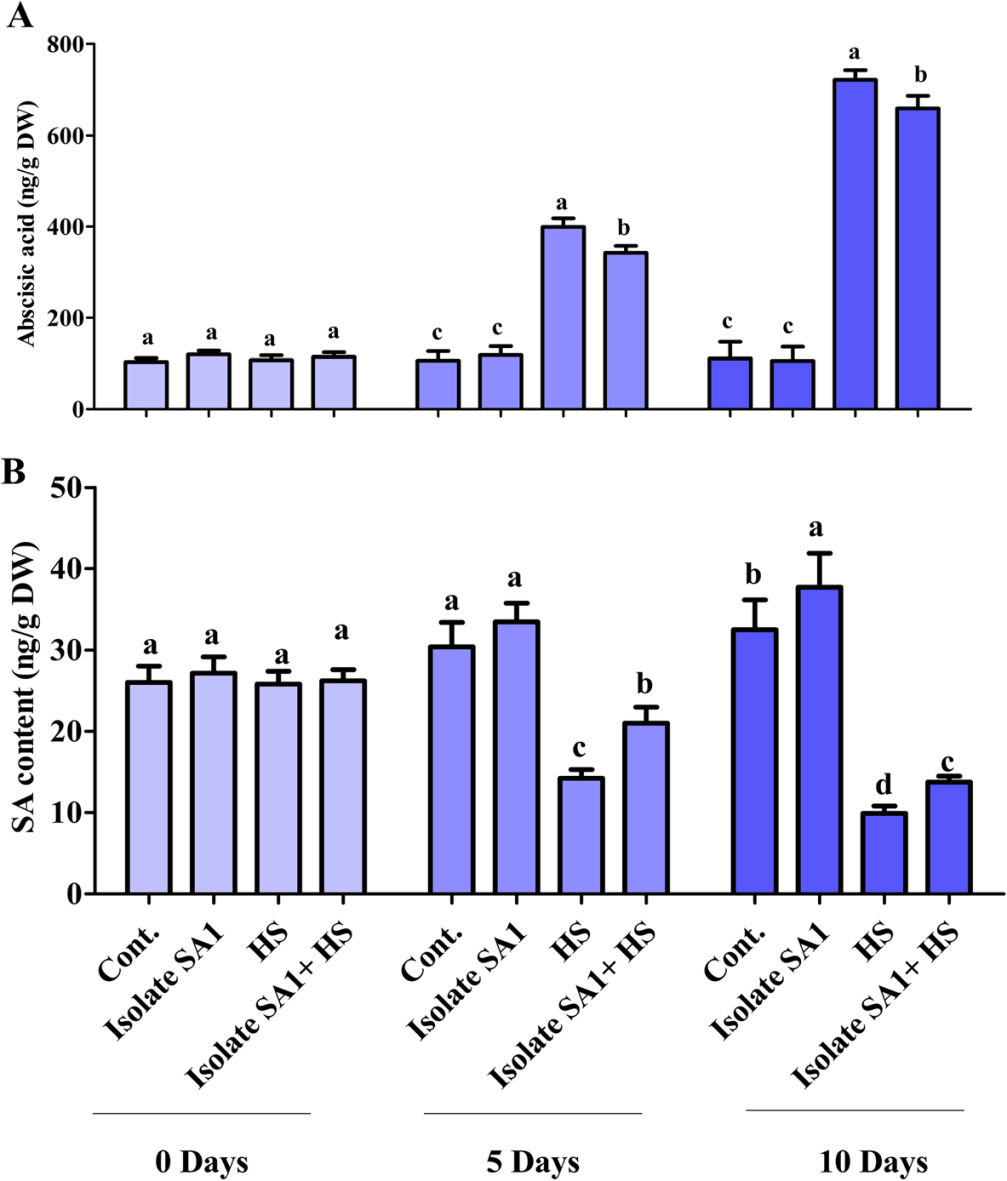
Endogenous abscisic acid (ABA) and salicylic acid (SA) quantification in SA1-inoculated soybean plants. **a** ABA and **b** SA contents under heat stress were determined at different time points (0, 5, and 10 days). Each data point is the mean of at least three replicates. Error bars represent standard errors. The bars with different letters are significantly different from each other as evaluated by DMRT analysis

There was no significant difference in the endogenous ABA and SA content of SA1-inoculated soybean plants compared with control soybean plants. However, when the soybean plants were exposed to heat stress (5 and 10 days), ABA was increased, and SA was decreased. Endogenous ABA was significantly increased by 3-folds and 7-folds after 5 and 10 days of heat stress, respectively (Fig. 4a). Inoculation with the thermotolerant isolate SA1 markedly reduced ABA to 2.1-folds and 5.4-folds, respectively. In contrast to endogenous ABA, a decrease in the SA of soybean plants exposed to heat stress was observed after 5 and 10 days. Endogenous SA was significantly reduced from 53 to 67% after 5 and 10 days; however, inoculation with SA1 augmented the heat stress response and reduced the SA content from 30 to 64% (Fig. 4b).

### Antioxidant enzyme regulation during heat stress

**Fig. 5:**
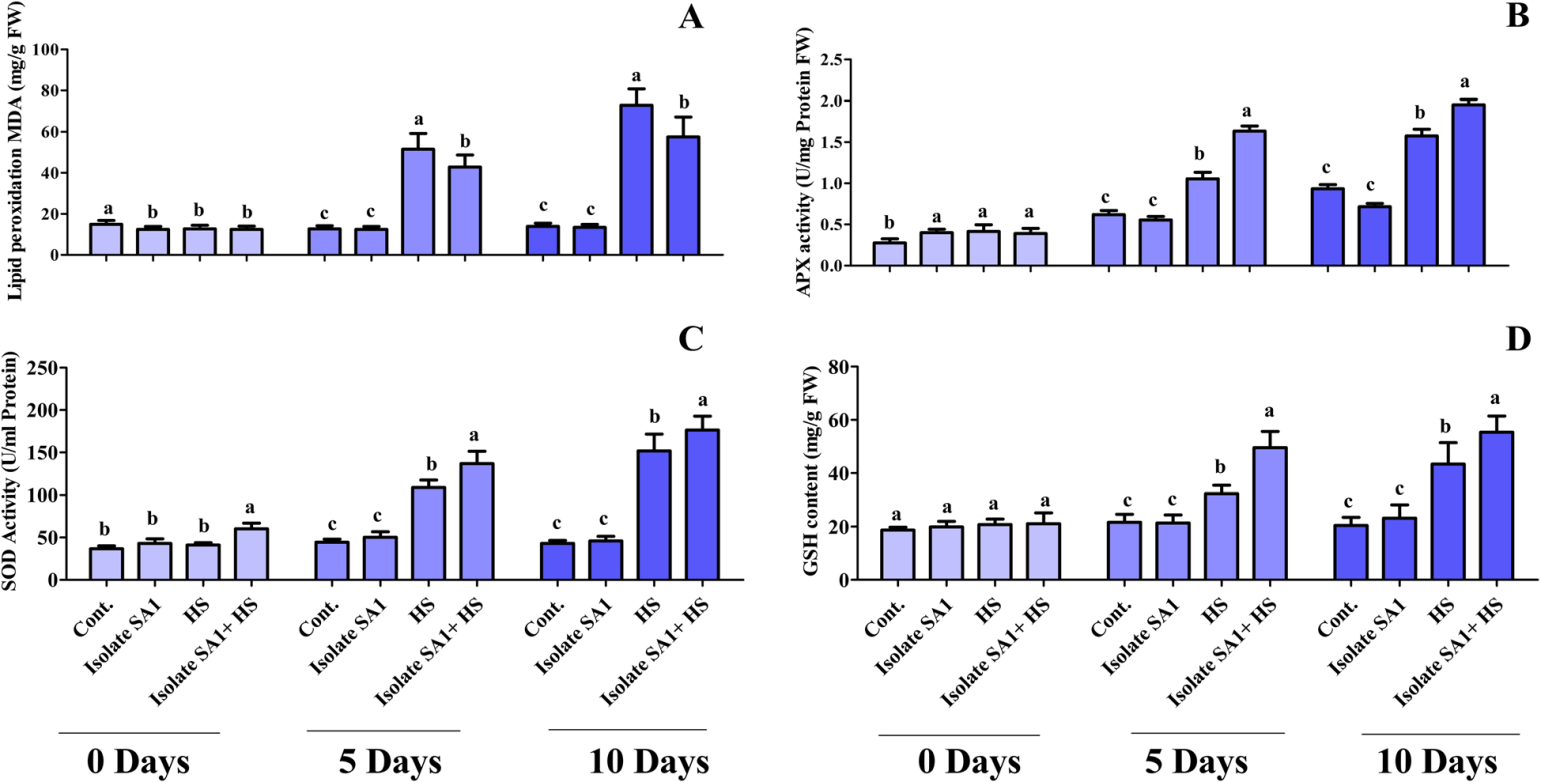
Effect of heat stress on antioxidant components. **a** Lipid peroxidation (LPO), **b** ascorbic acid peroxidase (APX), **c** superoxide dismutase (SOD), and **d** reduced glutathione (GSH) contents in soybean plants with and without SA1 inoculation were determined at different time points (0, 5, and 10 days). Each data point is the mean of three replicates. Error bars represent standard errors. The bars with different letters are significantly different from each other as evaluated by DMRT analysis

To assess the extent of LPO, the malondialdehyde (MDA) content in soybean plants under normal and heat stress conditions was evaluated. Heat stress led to the generation of MDA, which in turn led to the induction of LPO. The results revealed that there was no significant difference in the MDA content of inoculated and non-inoculated soybean plants under controlled conditions. However, under heat stress, a higher MDA level was observed in non-inoculated plants (4.3-fold) compared with SA1-inoculated plants (3.5-fold) after 5 days. A similar pattern was observed after 10 days for soybean plants inoculated with SA1 and exposed to heat stress (Fig. 5a). Similarly, the APX content of inoculated and non-inoculated plants was different. Heat stress for 5 and 10 days induced an increase in APX up to 59 and 120%, respectively, in non-inoculated soybean plants. However, SA1 inoculation resulted in a considerable increase in APX after 5 and 10 days (3-fold and 4-fold, respectively) (Fig. 5b). In addition, SOD and GSH analysis results revealed an increase in the SOD and GSH contents of soybean plants exposed to heat stress and those inoculated with SA1 compared with normal control plants. Soybean plants exposed to heat stress had enhanced SOD (2.6-fold) and GSH (49%) contents after 5 days. However, inoculation with SA1 greatly increased SOD (3.1-fold) and GSH (129%) contents. A similar pattern was observed after 10 days for soybean plants inoculated with SA1 and exposed to heat stress (Fig. 5c&d).

### Effect of heat stress and PGPEB on amino acid synthesis

Amino acid contents were determined to analyze the regulation of physiological functions under normal and heat stress conditions with SA1 inoculation. Under normal conditions, SA1 inoculation increased aspartic acid (1.30%), glutamic acid (3.73%), and proline (3.11%) and slightly decreased alanine (0.23%), phenylalanine (1.02%), and arginine (0.84%). For non-inoculated plants under heat stress for 5 and 10 days, glutamic acid (27.82 and 38.69%, respectively), alanine (15 and 28%, respectively), and phenylalanine (18.97 and 48.55%, respectively) were decreased, and only aspartic acid (2.07 and 8.09%, respectively) and proline (26.58 and 56.18%, respectively) were higher. However, under heat stress for 5 and 10 days, the amino acids aspartic acid (8.23 and 10.57%, respectively), glutamic acid (9.57 and 11.38%, respectively), alanine (9.28 and 11.51%, respectively), phenylalanine (12.09 and 11.81%, respectively), arginine (36.56 and 30.25%, respectively), and proline (41 and 85%, respectively) were increased in SA1-inoculated plants compared with non-inoculated plants (Table 3).

**Table 3:** Quantification of different amino acids at different time points (0, 5 and 10 Days). Each data point is the mean of at least three replicates. Error bars represent standard errors. The bars represented with different letters are significantly different from each other as evaluated by DMRT

	Aspartic acid	Glutamic acid	Alanine	Phenylalanine	Arginine	Proline
**0 days**						
**Cont.**	29.89 ± 4.6^a^	22.46 ± 2.5^a^	12.49 ± 1.5^a^	10.75 ± 1.3^a^	10.61 ± 2.3^ab^	12.42 ± 1.6^a^
**Isolate SA1**	30.28 ± 2.7^a^	23.30 ± 2.7^a^	12.47 ± 1.6^a^	10.64 ± 1.9^a^	10.52 ± 1.4^ab^	12.90 ± 1.8^a^
**HS**	28.93 ± 3.5^a^	22.97 ± 2.2^a^	13.85 ± 2^a^	09.37 ± 1.1^a^	10.00 ± 1.5^b^	13.81 ± 2.1^a^
**HS + SA1**	29.54 ± 3.8^a^	23.01 ± 3.0^a^	12.73 ± 2.0^a^	11.25 ± 2.2^a^	11.74 ± 1.6^a^	12.14 ± 2.3^a^
**5 days**						
**Cont.**	30.50 ± 2.9^c^	21.44 ± 2.7^a^	12.07 ± 2.3^ab^	8.06 ± 1.5^c^	10.60 ± 1.0^b^	13.99 ± 1.7^c^
**Isolate SA1**	31.13 ± 4.4^c^	21.58 ± 3.5^a^	12.94 ± 1.8^a^	10.01 ± 1.7^a^	11.84 ± 2.1^b^	13.39 ± 1.6^c^
**5 day HS**	33.05 ± 1.6^b^	16.96 ± 1.4^b^	10.89 ± 1.9^b^	9.65 ± 1.7^b^	13.94 ± 2.5^a^	15.76 ± 2.2^b^
**5 day HS+ SA1**	35.05 ± 2.5^a^	20.39 ± 3.0^a^	11.22 ± 2.5^ab^	9.31 ± 1.2^b^	13.70 ± 4.0^a^	17.59 ± 3.4^a^
**10 days**						
**Cont.**	32.33 ± 3.09^c^	20.99 ± 0.7^a^	11.66 ± 1.4^a^	9.76 ± 0.5^a^	11.65 ± 2.4^bc^	14.43 ± 2.1^c^
**Isolate SA1**	32.38 ± 4.8^c^	22.62 ± 3.9^a^	11.01 ± 0.2^a^	9.14 ± 2.9^ab^	10.32 ± 1.7^c^	13.56 ± 2.1^c^
**10 day HS**	36.14 ± 3.9^b^	13.49 ± 2.6^c^	9.91 ± 1.3^c^	8.13 ± 1.0^c^	12.99 ± 2.3^b^	19.48 ± 1.5^b^
**10 day HS+ SA1**	44.87 ± 5.3^a^	18.93 ± 1.6^b^	10.59 ± 1.2^b^	9.29 ± 1.1^b^	15.19 ± 1.7^a^	23.03 ± 2.8^a^

### Gene expression **during heat stress and bacterial inoculation**

**Fig. 6:**
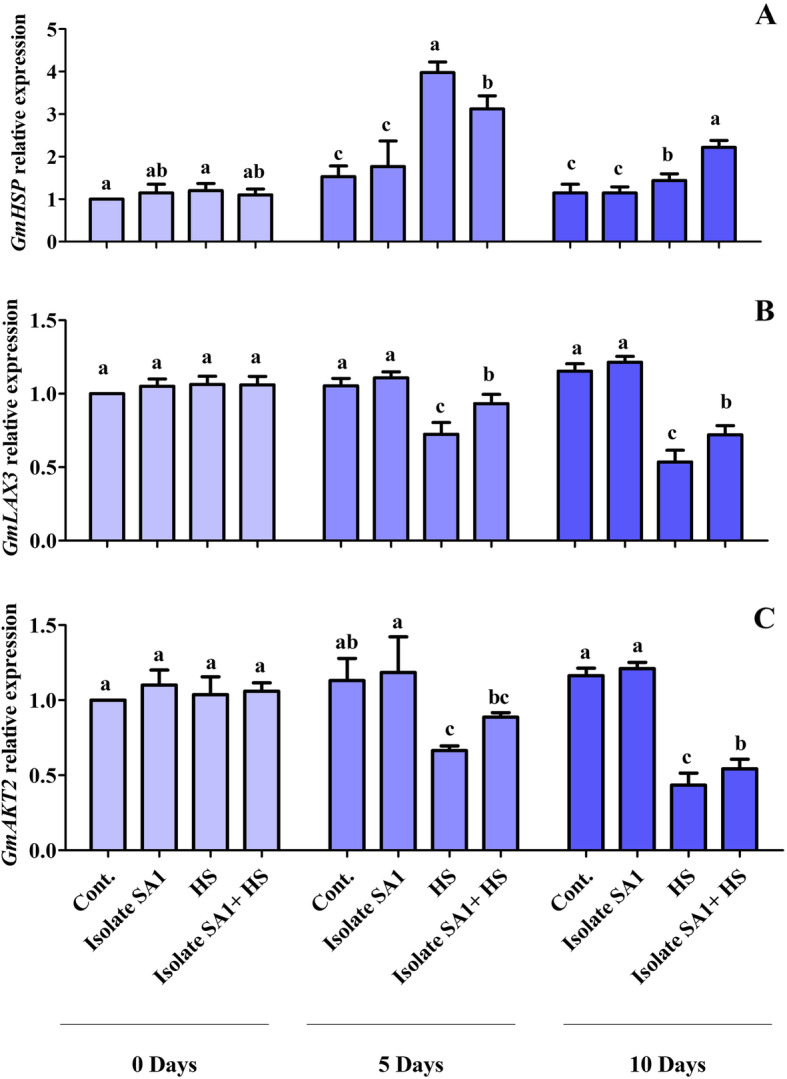
Expression of genes in soybean plants. The relative expression of genes in soybean plants with and without SA1 inoculation under heat stress was determined at different time points (0, 5, and 10 days). **a***GmHSP*, **b***GmLAX3*, and **c***GmAKT2*. The values were calculated relative to those of actin gene expression (the means of three replicates). Error bars represent standard errors. The bars with different letters are significantly different from each other as evaluated by DMRT analysis

The transcriptional level of HSPs in response to heat stress was evaluated. There was no significant difference in the *GmHSP* expression of SA1-inoculated soybean plants compared with control soybean plants. However, when non-inoculated plants were exposed to heat stress for 5 days, HSP expression was significantly increased (2.5folds) compared with the expression of control plants whereas inoculation with isolateSA1 markedly reduced HSP expression (2folds) after 5 days of heat stress. In contrast, HSP expression was reduced to 1 fold after 10 days of heat stress in non-inoculated plants, and inoculation with SA1 augmented the heat stress response and increased the expression of HSPs up to 1.9 folds compared with control plants (Fig. 6a). Similarly, the role of *GmLAX3* and *GmAKT2* was determined by analyzing their expression level in soybean plants under normal and heat stress conditions. Under normal conditions, there was no significant difference in *GmLAX3*; however, heat stress for 5 and 10 days decreased the expression of *GmLAX3* up to 0.68 and 0.5folds, respectively. On the other hand, inoculation with SA1 mitigated heat stress and increased the expression of *GmLAX3* to 0.88folds and 0.68folds after 5 and 10 days, respectively (Fig. 6b). The higher expression of *GmAKT2* markedly increased K^+^ levels in both control plants and heat-stressed plants inoculated with SA1. The expression of *GmAKT2* was increased up to 1.18folds and 1.2 folds after 5 and 10 days, respectively, in SA1-inoculated plants compared with control plants. After exposure to heat stress for 5 and 10 days, the expression of *GmAKT2* was decreased to 0.58 and 0.38 folds, respectively; however, inoculation with SA1 increased the expression up to 0.8folds and 0.47folds, respectively (Fig. 6c).

## Discussion

High temperatures directly affect the photosynthesis and transpiration process, consequently affecting Soybean yield [35]. Currently, the robust nutritional properties and different beneficial characteristics of soybean constituents have contributed to its increased demand worldwide. Therefore, there is a need to boost soybean production under heat stress conditions. Accordingly, there has been increased interest in the use of PGPEB as a promising alternative to alleviate plant heat stress, and the role of microbes in the management of abiotic and biotic stresses has been recognized. Based on plant growth-promoting traits and thermotolerant activities, SA1 was selected for the experiments with soybean plants under heat stress (Fig. 3; Table 1). Different studies have revealed that various strains in onion, barely, felty germander, and wheat [36–38] have plant growth-promoting traits and phytohormone production (IAA and GA). The isolate used in the present study showed the capability for IAA, GA, and organic acid production and phosphate solubilization (Fig. 2a&b). Organic acids are considered as an important source of carbon and are rich in energy. Our results revealed that the CF of isolate SA1 contained succinic acid, lactic acid, formic acid, and butyric acid (Fig. 2c). In addition, organic acids can enhance the degree and rate of metal dissolution, pH homeostasis, and plant growth [39]. Phosphate-solubilizing plant growth-promoting bacteria have been reported to produce organic acids, facilitating phosphorus and essential nutrient uptake from the soil [40, 41]. The microbes used in this study showed an innate ability to produce organic acids (Fig. 2c), and the phosphate-solubilizing activity of these microbes may affect the growth and development of the plants. Similar results have been reported by several researchers who found that organic acid-producing and phosphate-solubilizing bacteria mitigated the adverse effects of abiotic stress and enhanced plant growth [42–45].

Heat stress can affect the biochemical and molecular aspects of plant growth and lead to physiological and morphological disorders in plants [4–6]. In this study, heat stress was found to inhibit plant growth and development; however, inoculation with the thermotolerant isolate SA1 markedly alleviated the adverse effects of heat stress after 5 and 10 days (Table 1). Similar results were also reported by various studies showing that the use of thermotolerant plant growth-promoting bacteria could increase thermotolerance in sorghum, chickpea, tomato, potato, and canola plants [19–24]. Several plant species have evolved several mechanisms to guard the photosynthetic apparatus against damages from heat stress by encoding for the production of HSPs that bind to thylakoid membranes and protect the PSII complex and whole chain of electron transport [3, 6, 46, 47]. However, under severe heat stress, these protective mechanisms may be inadequate for ensuring plant viability. Our findings showed that when the plants were subjected to heat stress, a decrease in the *Chl a*, *Chl b*, carotenoid and chlorophyll fluorescence contents was observed; however, inoculation with SA1 mitigated heat stress and increased the chlorophyll and carotenoid contents (Table 2). Similar results were also reported by previous studies showing that the use of thermotolerant bacteria could increase the total chlorophyll content in wheat and canola plants under heat stress [19, 48]. The increase in the chlorophyll content may also be explained by the increased photosynthetic leaf area of plants inoculated with bacteria compared with that of non-inoculated plants, which was reduced due to heat stress [18–20, 23].

Phytohormones respond to changing environmental conditions by regulating plant growth and stress tolerance to maintain the viability of the plants. Previous studies have demonstrated that phytohormones are actively involved in the response to heat stress [49–51]. IAA is a key phytohormone that plays a vital role in the growth and developmental process of plants [52]. Additionally, IAA can mediate the tolerance of plants to heat stress through the activation of antioxidant enzymes, gene expression regulation, osmoprotectant (proline) synthesis, and enhancement of photosynthetic pigment accumulation [52]. IAA-producing bacteria have been found to increase the length and root surface of plants, and the plants have better access to available nutrients in the soil [17, 53]. In agreement with these findings, our bacterial isolate (SA1) was observed to produce IAA (Fig. 2b) and greatly mitigate the adverse effects of heat stress on soybean plants. GAs also play a prominent role in the alleviation of abiotic stress [54, 55]. Some studies have described the protective role of GAs in the plant adaptation to abiotic stresses and detoxification of heavy metals [54–58]. Stavang et al. [59] also reported that GAs play a role in the thermoperiodic response in peas. Microbes could produce GAs, which are involved in plant growth promotion. The capability for GA production is an important microbial trait that could greatly mitigate the adverse effects of heat stress on plants and promote their growth and development [17]. GAs have been reported to ameliorate heat stress and enhance plant growth and development [17, 59, 60]. Despite the existence of several forms of GAs, biologically active GAs are limited to GA_1_, GA_3_, and GA_4_ in different microorganisms [61, 62]. These biologically active GAs can promote plant growth by reducing stress hormones such as ABA [63–65]. As plants perceive stress conditions, they regulate stress hormones such as ABA through active chemical signals, which contribute to the extreme sensitivity of stomatal conductance [65, 66]. Plant-microbe interaction has been reported to mitigate the adverse effects of abiotic stress by reducing ABA levels [17, 18, 67]. Similar results were observed in our study where SA1 inoculation reduced ABA and increased plant growth parameters (Fig. 4a). Similarly, SA is another plant hormone that plays an important role in various physiological processes and biochemical reactions. According to Zhang et al. [68], in mutualistic plant-microbe interaction, SA can induce systemic resistance in plants. In agreement with this, Wang et al. [69] indicated that SA can ameliorate abiotic stress by inducing ROS generation. The accumulation of SA has been associated with heat stress tolerance in various plants including Kentucky Bluegrass [16], grapevine [70], potato [71], bean and tomato [72], *Arabidopsis* [73], and grape plants [74]. In the present study, SA1 inoculation greatly enhanced endogenous SA levels in soybean plants under heat stress and normal conditions (Fig. 4b). Our findings are in agreement with those of previous studies showing the ability of bacterial inoculums to enhance endogenous levels of SA and contribute to the growth and development of plants under abiotic stress [75, 76]. Furthermore, heat stress leads to the generation of ROS such as superoxide anion, H_2_O_2_, and singlet oxygen, which cause cellular toxicity and damage to cell structures in plants. However, plants may develop a complex antioxidant defense system consisting of MDA, SOD, glutathione reductase (GR), and APX, which can protect the plants against cellular stress, remove free radicals, and scavenge excess ROS. In comparison with non-inoculated soybean plants under heat stress, soybean plants inoculated with SA1 produced less ROS and exhibited an increase in antioxidant SOD, MDA, GR, and APX activity (Fig. 5). Similar results were reported by Ali et al. [20], Abd El-Daim et al. [22], and Park et al. [17], who found that PGPB could enhance the activity of different ROS-scavenging enzymes under heat stress.

Amino acids play a prominent role in the physiological and biochemical functions of plants by modulating membrane permeability, osmolytes, ion uptake, and enzymatic activity and enhancing abiotic stress tolerance [77, 78]. A rescue effect and significantly enhanced key amino acid contents were observed in soybean plants inoculated with SA1 compared with non-inoculated soybean plants under heat stress (Table 3). Several studies have demonstrated that an increase in amino acid contents could increase the tolerance to abiotic stress by inoculating plant growth-promoting bacteria [79–83]. Heat stress was found to decrease the accumulation of proline; however, proline levels were enhanced in SA1-inoculated soybean plants under control and heat stress conditions. Proline accumulation is considered as an adaptive mechanism under heat stress [84–87]. In addition, plants could alter the expression of heat-stress-dependent regulatory proteins such as heat shock transcription factors (HSFs) and HSPs, which may enhance tolerance and protect protein functions under heat stress. HSPs are essential for maintaining and restoring protein structures and stabilizing the condition of plants under heat stress [11, 88]. Islam et al. [22] reported the upregulated expression of HSPs; however, in inoculated wheat seedlings, a decrease in HSP transcript levels was observed during heat stress. Similar HSP transcriptional levels were observed in SA1-inoculated soybean plants after 5 days of heat stress; however, after 10 days, an opposite effect was observed (Fig. 6a). The induction of HSPs is usually accompanied by the induction of tolerance to heat and other stresses. Previous studies have revealed that HSPs could act as “molecular chaperones”, and their overexpression is well known to enhance thermotolerance [10, 12, 89]. Furthermore, genome-wide transcriptomic analysis of soybean revealed that some hormone-related genes were expressed differentially under abiotic stress. *GmLAX3* and *GmAKT2* are among the key genes involved in the regulation of the ABA-dependent pathway, decreasing ROS generation, and enhancing the transport of K^+^ under abiotic stress. The present results revealed that *GmLAX3* and *GmAKT2* were downregulated; however, SA1 stimulated the expression of *GmLAX3* and *GmAKT2* under heat stress after 5 and 10 days (Fig. 6b&c). Similar results were also reported by previous studies showing that that *GmLAX3* and *GmAKT2* may be involved in the plant stress response by modulating auxin and ABA stimuli, decreasing ROS generation, and enhancing potassium gradients, which are critical in plant vascular tissues under abiotic stress [90–92].

## Conclusion

In conclusion, the present study demonstrated the ability of *B. cereus* SA1 to produce biologically active metabolites such as GAs, IAA, and organic acids. *B. cereus* SA1 inoculation enhanced the chlorophyll fluorescence and photosynthetic pigments of soybean plants under normal growth conditions as well as under heat stress. This improvement in plant growth was coupled with changes in the endogenous levels of several phytohormones (ABA and SA), essential amino acids, and *GmHSP*, *GmLAX3*, and *GmAKT2* expression. Active plant growth-promoting strains such as SA1 could be used to develop biofertilizers, and further studies with a focus on HSPs are needed to understand their function in heat stress tolerance in soybean plants.

## Methods

### Isolation and screening of endophytic bacterial strains

For isolation of endophytic bacteria, we collect four plants samples (*Artemisia princeps*, *Chenopodium ficifolium*, *Oenothera biennis*, and *Echinochloa crus-galli*) from the sand dunes at Pohang beach, Republic of Korea as preciously described by Khan et al. [27, 93]. Furthermore all isolate were screened for the production of indole-3-acetic acid (IAA) using Salkowski reagent [94], phosphate solubilization potential using trypticase soy agar medium supplemented with Ca_3_(PO_4_)_2_ [95] and siderophores production using Chrome Azurol S (CAS) agar plates [96]. For bioassay assessment, the selected bacterial isolates, which revealed distinct plant growth-promoting traits in the initial screening, were also screened with *Waito-C* (GA-deficient) rice seedlings. The test seeds were grown using Hoagland solution for 14 consecutive days under controlled environmental conditions [97].

### Screening for thermotolerant and molecular identification

For thermotolerant screening; multiple PGP traits producing bacteria were grown in LB media at 30 °C, 35 °C, 40 °C, and 45 °C in rotary shaker for 6 days and there growth was recorded using a spectrophotometer (600 nm). For molecular identification, the method of Sambrook and Russell [98] was used for genomic DNA extraction and 16S rRNA-specific primers were used and amplified according to the protocol of Khan et al. [93]. The NCBI BLAST program was used to determine nucleotide sequence homology of selected isolate, and MEGA 6.1 software was used for phylogenetic analysis [99].

### Quantification of in vitro IAA, gibberellin (GA), and organic acid production of isolate SA1

Isolate SA1 was grown in LB media and centrifuged at 5000×*g* for the quantification of IAA, GA, and organic acid production. Following the method of Khan et al. [100], the culture broth was analyzed for IAA production through GC/MS-SIM. Similarly for GA content, a previous method of Khan et al. [101] was used, and the data were calculated as ng per ml. For organic acid analysis, the method of Kang et al. [102] was used by centrifuging the culture broth of isolate SA1, the supernatant was filtered through a 0.22 μm Millipore filter paper and 10 μl sample was injected into a high-performance liquid chromatography (HPLC) system (Waters 600; Waters Corp., Milford, MA, USA). All of the samples were analyzed in triplicate.

### Plant-microbe growth and heat stress conditions

Soybean seeds (variety, Pungsannamul) were collected from the Soybean Genetic Resource Center at Kyungpook National University (Daegu, Republic of Korea) and tested for viability. For surface sterilization of the seeds, sodium hypochlorite (2.5% for 30 min) was used followed by rinsing with sterilized distilled water. After 10 days of germination in trays, uniform seedlings were selected and were grown individually in a sterilized plastic pots (10 cm × 9 cm) at V1 stage. Plastic pots filled with autoclaved horticulture soil consisting of peat moss (10–15%), zeolite (6–8%), coco peat (45–50%), perlite (35–40%), NH_4_^+^ (∼0.09 mg/g), NO_3_^−^ (∼0.205 mg/g), P_2_O_5_ (∼0.35 mg/g), and K_2_O (∼0.1 mg/g) were used for the growth of soybean plants. Two days after transplantation, 50 ml of freshly diluted bacterial culture (10^9^ CFU/mL) was inoculated to each pot; this was repeated further two time after 5 days, while autoclaved double distilled water were used for control soybean plants. At V3 stage plants were exposed to heat stress and plants sample were collected at 0 day, 5 days and 10 days. The experimental design included the following groups: (a) control (normal soybean), (b) soybean with SA1, (c) soybean with 5-day heat stress treatment with or without SA1, (d) soybean with 5-day heat stress treatment with or without SA1, and (e) soybean with 10-day heat stress treatment with or without SA1 in a growth chamber (for normal soybean plants: 24 h cycles consisting of 28 °C for 14 h and 25 °C for 10 h with a relative humidity of 60–70%; for heat stress plants: 24 h cycles consisting of 40 °C for 14 h and 30 °C for 10 h with a relative humidity of 60–70%). After stress completion, growth attributes (shoot and root length, fresh and dry weight, and chlorophyll content) were recorded, and the plants were immediately harvested in liquid nitrogen and stored at − 80 °C until further biochemical analyses. For chlorophyll estimation, 500 mg of soybean leaves were ground, and the photosynthetic pigment was extracted with acetone (80%). The contents of chlorophyll a and b (*Chl a* and *Chl b*) were estimated as described by Khan et al. [100] using a spectrophotometer with an absorbance of 663 nm and 465 nm. The total carotenoid was estimated at 480 nm as described by Vimala and Poonghuzhali [103]. A chlorophyll fluorometer (FIM 1500; ADC Bioscientific Ltd., Hoddesdon, UK) was used for the measurement of chlorophyll fluorescence. The efficiency of photosystem II (Fv/Fm) was calculated as described by Genty [104].

### Plant endogenous phytohormone quantification

The endogenous phytohormones of plant samples were analyzed and quantified in a controlled environment. Endogenous ABA was quantified according to the method of Khan et al. [105]. On the other hand, SA was quantified following the method of Seskar et al. [106]. Freeze-dried aerial parts were quantified using a HPLC system equipped with a fluorescence detector (excitation and emission at 3005 and 365 nm, respectively; Shimadzu RF-10AXL; Shimadzu, Kyoto, Japan) and fitted with a C18 reverse phase HPLC column (particle size 5 m, pore size 120 Å; HP Hypersil ODS; Waters Co., Milford, MA, USA) at a defined flow rate (1.0 ml/min).

### Antioxidant analysis

Lipid peroxidation (LPO) in soybean were measured spectrophotometrically according to the method of Khan et al. [107]. Similarly the activity of APX in soybean was measured following the method of Kim et al. [108]. The method of Marklund and Marklund [109] was adapted for SOD activity assay. To determine the reduction in GSH concentration, the detail method of Ellman [110] and Asaf et al. [111] were used. All experiments were performed in triplicate.

### RNA extraction, cDNA synthesis, and qRT-PCR analysis

Total RNA was extracted from soybean leaves using TRIzolTM reagent following the protocol of Chan et al. [112]. RNA quality was examined by nano drop machine; while qPCRIBO cDNA synthesis Kit was used for cDNA synthesis. qRT-PCR were performed using qPCRBIO SYBR Green Kit as described previously by Jan et al. [113], and actin was used as a reference gene. A 20 μl volume was maintained by using 7 μl ddH2O, 1 μl primer, 10 μl SYBR green and 1 μl DNA templet for each reaction (Supplementary Table 2).

### Amino acid quantification

The method of Asaf et al. [111] was used for amino acid analysis. Frieze-dried plant samples (100 mg) were hydrolyzed under vacuum in 6 N HCl in an ampulla tube for 24 h at 110 °C followed by 24 h at 80 °C. The solid residues were homogenized in 0.02 N HCl and filtered with a 0.45 m filter membrane. The amino acids were then analyzed using an automated amino acid analyzer (L-8900; Hitachi, Tokyo, Japan). The concentration was determined by comparison with specific standards.

### Statistical analysis

The experiments were performed in triplicate, and the obtained results were used for further analysis. The difference among the mean values was compared with Duncan’s multiple range test (DMRT) using SAS 9.2 statistical software (SAS Institute, Cary, NC, USA). The graphical presentation was made in GraphPad Prism.

## Supplementary information

**Additional file 1: Figure S1.** Growth of multiple plant growth-promoting traits producing endophytic bacteria (PGPEB). PGPEB were grown in LB media at 25 °C, 30 °C, 35 °C, 40 °C, and 45 °C for 6 days, and the growth was examined using a spectrophotometer at 600 nm. Each data point is the mean of three replicates.

**Additional file 2: Figure S2.** Growth of isolate SA1 in LB media. Isolate SA1 were grown in LB media at 25 °C, 30 °C, 35 °C, 40 °C, and 45 °C for 36 h, and the growth was examined using a spectrophotometer at 600 nm. Each data point is the mean of three replicates.

**Additional file 3: Table S1.** Description of plants species and number of their yielded endophytic isolates. The isolates were preliminary sorted for single or multiple plant growth beneficial activities.

**Additional file 4: Table S2.** List of primers used in this study

## Data Availability

The dataset generated or analyzed and strain used in the current study were submitted to NCBI with the GenBank accession no. MH032605 are included in this published article.

## Abbreviation

PGPEB: Plant growth-promoting endophytic bacteria
GA: Gibberellin
IAA: Indole-3-acetic acid
ABA: Abscisic acid
SA: Salicylic acid
APX: Ascorbic acid peroxidase
SOD: Superoxide dismutase
GSH: Glutathione
HSP: Heat shock protein
*GmAKT2*: Potassium channel AKT2

## References

[1] Lobell DB, Gourdji SM (2012). The influence of climate change on global crop productivity. Plant Physiol.

[2] Parry ML, Canziani OF, Palutikof JP, van der Linden PJ, Hanson CE, IPCC (2007). Climate Change 2007: Impacts, Adaptation and Vulnerability. Contribution of Working Group II to the Fourth Assessment Report of the Intergovernmental Panel on Climate Change.

[3] Ortiz R, Sayre KD, Govaerts B, Gupta R, Subbarao GV, Ban T, Hodson D, Dixon JM, Iván Ortiz-Monasterio J, Reynolds M (2008). Climate change: can wheat beat the heat?. Agric Ecosystems Environ.

[4] Bita C, Gerats T (2013). Plant tolerance to high temperature in a changing environment: scientific fundamentals and production of heat stress-tolerant crops. Front Plant Sci.

[5] Siddiqui MH, Al-Khaishany MY, Al-Qutami MA, Al-Whaibi MH, Grover A, Ali HM, Al-Wahibi MS (2015). Morphological and physiological characterization of different genotypes of faba bean under heat stress. Saudi J Biol Sci.

[6] Hasanuzzaman M, Nahar K, Alam MM, Roychowdhury R, Fujita M (2013). Physiological, biochemical, and molecular mechanisms of heat stress tolerance in plants. Int J Mol Sci.

[7] Awasthi R, Bhandari K, Nayyar H (2015). Temperature stress and redox homeostasis in agricultural crops. Front Environ Sci.

[8] Grene R (2002). Oxidative stress and acclimation mechanisms in plants. Arabidopsis Book.

[9] Hasanuzzaman M, Hossain MA, da Silva JAT, Fujita M, Venkateswarlu B, Shanker AK, Shanker C, Maheswari M (2012). Plant Response and Tolerance to Abiotic Oxidative Stress: Antioxidant Defense Is a Key Factor. Crop Stress and its Management: Perspectives and Strategies.

[10] Yildiz M, Terzi H (2008). Small heat shock protein responses in leaf tissues of wheat cultivars with different heat susceptibility. Biologia.

[11] Xu Z-S, Li Z-Y, Chen Y, Chen M, Li L-C, Ma Y-Z (2012). Heat shock protein 90 in plants: molecular mechanisms and roles in stress responses. Int J Mol Sci.

[12] Queitsch C, Hong S-W, Vierling E, Lindquist S (2000). Heat shock protein 101 plays a crucial role in Thermotolerance in Arabidopsis. Plant Cell.

[13] Hasanuzzaman M, Bhuyan MHMB, Nahar K, Hossain MS, Mahmud JA, Hossen MS, Masud AAC, Moumita FM (2018). Potassium: a vital regulator of plant responses and tolerance to abiotic stresses. Agronomy.

[14] Jan N, Qazi HA, Ramzan S, John R, Gosal SS, Wani SH (2018). Developing Stress-Tolerant Plants Through In Vitro Tissue Culture: Family Brassicaceae. Biotechnologies of Crop Improvement, Volume 1: Cellular Approaches.

[15] Lamaoui M, Jemo M, Datla R, Bekkaoui F (2018). Heat and drought stresses in crops and approaches for their mitigation. Front Chem.

[16] He Y, Liu Y, Cao W, Huai M, Xu B, Huang B (2005). Effects of salicylic acid on heat tolerance associated with antioxidant metabolism in Kentucky bluegrass. Crop Sci.

[17] Park Y-G, Mun B-G, Kang S-M, Hussain A, Shahzad R, Seo C-W, Kim A-Y, Lee S-U, Oh KY, Lee DY (2017). Bacillus aryabhattai SRB02 tolerates oxidative and nitrosative stress and promotes the growth of soybean by modulating the production of phytohormones. PLoS One.

[18] Tiwari S, Prasad V, Chauhan PS, Lata C (2017). *Bacillus amyloliquefaciens* Confers Tolerance to Various Abiotic Stresses and Modulates Plant Response to Phytohormones through Osmoprotection and Gene Expression Regulation in Rice. Front Plant Sci.

[19] Ali SZ, Sandhya V, Grover M, Kishore N, Rao LV, Venkateswarlu B (2009). Pseudomonas sp. strain AKM-P6 enhances tolerance of sorghum seedlings to elevated temperatures. Biol Fertil Soils.

[20] Ali SZ, Sandhya V, Grover M, Linga VR, Bandi V (2011). Effect of inoculation with a thermotolerant plant growth promoting Pseudomonas putida strain AKMP7 on growth of wheat (Triticum spp.) under heat stress. J Plant Interact.

[21] Srivastava S, Yadav A, Seem K, Mishra S, Chaudhary V, Nautiyal CS (2008). Effect of high temperature on Pseudomonas putida NBRI0987 biofilm formation and expression of stress sigma factor RpoS. Curr Microbiol.

[22] Abd El-Daim IA, Bejai S, Meijer J (2014). Improved heat stress tolerance of wheat seedlings by bacterial seed treatment. Plant Soil.

[23] Issa A, Esmaeel Q, Sanchez L, Courteaux B, Guise J-F, Gibon Y, Ballias P, Clément C, Jacquard C, Vaillant-Gaveau N (2018). Impacts of Paraburkholderia phytofirmans strain PsJN on tomato (Lycopersicon esculentum L.) under high temperature. Front Plant Sci.

[24] Bensalim S, Nowak J, Asiedu SK (1998). A plant growth promoting rhizobacterium and temperature effects on performance of 18 clones of potato. Am J Potato Res.

[25] Egamberdieva D, Wirth SJ, Alqarawi AA, Abd Allah EF, Hashem A (2017). Phytohormones and Beneficial Microbes: Essential Components for Plants to Balance Stress and Fitness. Front Microbiol.

[26] Khurshid H, Jan S, Baig D, Arshad M, Khan M (2017). Miracle crop: the present and future of soybean production in Pakistan. MOJ Biol Med.

[27] Khan MA, Asaf S, Khan AL, Ullah I, Ali S, Kang S-M, Lee I-J (2019). Alleviation of salt stress response in soybean plants with the endophytic bacterial isolate Curtobacterium sp. SAK1. Ann Microbiol.

[28] Khan MA, Ullah I, Waqas M, Hamayun M, Khan AL, Asaf S, Kang S-M, Kim K-M, Jan R, Lee I-J (2019). Halo-tolerant rhizospheric Arthrobacter woluwensis AK1 mitigates salt stress and induces physio-hormonal changes and expression of GmST1 and GmLAX3 in soybean. Symbiosis.

[29] Jooyandeh H (2011). Soy products as healthy and functional foods. Middle-East J Sci Res.

[30] Ghani M, Kulkarni KP, Song JT, Shannon JG, Lee J-D (2016). Soybean sprouts: a review of nutrient composition, health benefits and genetic variation. Plant Breeding Biotechnol.

[31] Chebrolu KK, Fritschi FB, Ye S, Krishnan HB, Smith JR, Gillman JD (2016). Impact of heat stress during seed development on soybean seed metabolome. Metabolomics.

[32] Sgobba A, Paradiso A, Dipierro S, De Gara L, de Pinto MC (2015). Changes in antioxidants are critical in determining cell responses to short- and long-term heat stress. Physiol Plant.

[33] Thuzar M, Puteh AB, Abdullah NAP, Mohd Lassim MB, Jusoff K (2010). The effects of temperature stress on the quality and yield of soya bean [(Glycine max L.) Merrill.]. J Agric Sci.

[34] Zhao C, Liu B, Piao S, Wang X, Lobell DB, Huang Y, Huang M, Yao Y, Bassu S, Ciais P (2017). Temperature increase reduces global yields of major crops in four independent estimates. Proc Natl Acad Sci.

[35] Sehgal A, Sita K, Siddique KHM, Kumar R, Bhogireddy S, Varshney RK, HanumanthaRao B, Nair RM, Prasad PVV, Nayyar H (2018). Drought or/and Heat-Stress Effects on Seed Filling in Food Crops: Impacts on Functional Biochemistry, Seed Yields, and Nutritional Quality. Front Plant Sci.

[36] Kim WI, Cho WK, Kim SN, Chu H, Ryu KY, Yun JC, Park CS (2011). Genetic diversity of cultivable plant growth-promoting rhizobacteria in Korea. J Microbiol Biotechnol.

[37] Hassan SE-D (2017). Plant growth-promoting activities for bacterial and fungal endophytes isolated from medicinal plant of Teucrium polium L. J Adv Res.

[38] Mohite B (2013). Isolation and characterization of indole acetic acid (IAA) producing bacteria from rhizospheric soil and its effect on plant growth. J Soil Sci Plant Nutr.

[39] Jones D, Dennis P, Owen A, Van Hees P (2003). Organic acid behavior in soils–misconceptions and knowledge gaps. Plant Soil.

[40] Deubel A, Merbach W, Buscot F, Varma A (2005). Microorganisms in soils: roles in genesis and functions. Influence of Microorganisms on Phosphorus Bioavailability in Soils.

[41] Othman R, Panhwar QA, Khan MS, Zaidi A, Musarrat J (2014). Phosphate-Solubilizing Bacteria Improves Nutrient Uptake in Aerobic Rice. Phosphate Solubilizing Microorganisms: Principles and Application of Microphos Technology.

[42] Iqbal U, Jamil N, Ali I, Hasnain S (2010). Effect of zinc-phosphate-solubilizing bacterial isolates on growth of Vigna radiata. Ann Microbiol.

[43] Rodriguez H, Fraga R (1999). Phosphate solubilizing bacteria and their role in plant growth promotion. Biotechnol Adv.

[44] Pérez E, Sulbarán M, Ball MM, Yarzábal LA (2007). Isolation and characterization of mineral phosphate-solubilizing bacteria naturally colonizing a limonitic crust in the south-eastern Venezuelan region. Soil Biol Biochem.

[45] Verma P, Yadav AN, Khannam KS, Kumar S, Saxena AK, Suman A (2016). Molecular diversity and multifarious plant growth promoting attributes of bacilli associated with wheat (Triticum aestivum L.) rhizosphere from six diverse agro-ecological zones of India. J Basic Microbiol.

[46] Sharkey TD (2005). Effects of moderate heat stress on photosynthesis: importance of thylakoid reactions, rubisco deactivation, reactive oxygen species, and thermotolerance provided by isoprene. Plant Cell Environ.

[47] Ahammed GJ, Xu W, Liu A, Chen S (2018). COMT1 Silencing Aggravates Heat Stress-Induced Reduction in Photosynthesis by Decreasing Chlorophyll Content, Photosystem II Activity, and Electron Transport Efficiency in Tomato. Front Plant Sci.

[48] Glick BR, Liu C, Ghosh S, Dumbroff EB (1997). Early development of canola seedlings in the presence of the plant growth-promoting rhizobacterium Pseudomonas putida GR12-2. Soil Biol Biochem.

[49] Dobrá J, Černý M, Štorchová H, Dobrev P, Skalák J, Jedelský PL, Lukšanová H, Gaudinová A, Pešek B, Malbeck J (2015). The impact of heat stress targeting on the hormonal and transcriptomic response in Arabidopsis. Plant Sci.

[50] Peleg Z, Blumwald E (2011). Hormone balance and abiotic stress tolerance in crop plants. Curr Opin Plant Biol.

[51] Ahammed GJ, Li X, Zhou J, Zhou Y-H, Yu J-Q, Ahammed GJ, Yu J-Q (2016). Role of Hormones in Plant Adaptation to Heat Stress. Plant Hormones under Challenging Environmental Factors.

[52] Siddiqui MH, Alamri SA, Al-Khaishany MYY, Al-Qutami MA, Ali HM, Khan MN (2017). Sodium nitroprusside and indole acetic acid improve the tolerance of tomato plants to heat stress by protecting against DNA damage. J Plant Interact.

[53] Yang J, Kloepper JW, Ryu C-M (2009). Rhizosphere bacteria help plants tolerate abiotic stress. Trends Plant Sci.

[54] Vettakkorumakankav NN, Falk D, Saxena P, Fletcher RA (1999). A crucial role for gibberellins in stress protection of plants. Plant Cell Physiol.

[55] Tuna AL, Kaya C, Dikilitas M, Higgs D (2008). The combined effects of gibberellic acid and salinity on some antioxidant enzyme activities, plant growth parameters and nutritional status in maize plants. Environ Exp Bot.

[56] Maggio A, Barbieri G, Raimondi G, De Pascale S (2010). Contrasting effects of GA3 treatments on tomato plants exposed to increasing salinity. J Plant Growth Regul.

[57] Ashraf M, Karim F, Rasul E (2002). Interactive effects of gibberellic acid (GA3) and salt stress on growth, ion accumulation and photosynthetic capacity of two spring wheat (Triticum aestivum L.) cultivars differing in salt tolerance. Plant Growth Regul.

[58] Hisamatsu T, Koshioka M, Kubota S, Fujime Y, King RW, Mander LN (2000). The role of gibberellin biosynthesis in the control of growth and flowering in Matthiola incana. Physiol Plant.

[59] Stavang JA, Lindgård B, Erntsen A, Lid SE, Moe R, Olsen JE (2005). Thermoperiodic stem elongation involves transcriptional regulation of gibberellin deactivation in pea. Plant Physiol.

[60] Alonso-Ramírez A, Rodríguez D, Reyes D, Jiménez JA, Nicolás G, López-Climent M, Gómez-Cadenas A, Nicolás C (2009). Evidence for a role of gibberellins in salicylic acid-modulated early plant responses to abiotic stress in Arabidopsis seeds. Plant Physiol.

[61] Lee K-E, Adhikari A, Kang S-M, You Y-H, Joo G-J, Kim J-H, Kim S-J, Lee I-J (2019). Isolation and characterization of the high silicate and phosphate solubilizing novel strain Enterobacter ludwigii GAK2 that promotes growth in Rice plants. Agronomy.

[62] Bottini R, Cassan F, Piccoli P (2004). Gibberellin production by bacteria and its involvement in plant growth promotion and yield increase. Appl Microbiol Biotechnol.

[63] Zhang HJ, Zhang N, Yang RC, Wang L, Sun QQ, Li DB, Cao YY, Weeda S, Zhao B, Ren S (2014). Melatonin promotes seed germination under high salinity by regulating antioxidant systems, ABA and GA 4 interaction in cucumber (C ucumis sativus L.). J Pineal Res.

[64] Bashar KK, Tareq MZ, Amin MR, Honi U, Tahjib-Ul-Arif M, Sadat MA, Hossen QMM (2019). Phytohormone-mediated Stomatal response, escape and quiescence strategies in plants under flooding stress. Agronomy.

[65] Verma V, Ravindran P, Kumar PP (2016). Plant hormone-mediated regulation of stress responses. BMC Plant Biol.

[66] de Ollas C, Dodd IC (2016). Physiological impacts of ABA–JA interactions under water-limitation. Plant Mol Biol.

[67] Curá JA, Franz DR, Filosofía JE, Balestrasse KB, Burgueño LE (2017). Inoculation with Azospirillum sp. and Herbaspirillum sp. Bacteria Increases the Tolerance of Maize to Drought Stress. Microorganisms.

[68] Zhang S, Moyne A-L, Reddy MS, Kloepper JW (2002). The role of salicylic acid in induced systemic resistance elicited by plant growth-promoting rhizobacteria against blue mold of tobacco. Biol Control.

[69] Wang Q-J, Sun H, Dong Q-L, Sun T-Y, Jin Z-X, Hao Y-J, Yao Y-X (2016). The enhancement of tolerance to salt and cold stresses by modifying the redox state and salicylic acid content via the cytosolic malate dehydrogenase gene in transgenic apple plants. Plant Biotechnol J.

[70] Wang LJ, Fan L, Loescher W, Duan W, Liu GJ, Cheng JS, Luo HB, Li SH (2010). Salicylic acid alleviates decreases in photosynthesis under heat stress and accelerates recovery in grapevine leaves. BMC Plant Biol.

[71] Lopez-Delgado H, Dat JF, Foyer CH, Scott IM (1998). Induction of thermotolerance in potato microplants by acetylsalicylic acid and H2O2. J Exp Bot.

[72] Senaratna T, Touchell D, Bunn E, Dixon K (2000). Acetyl salicylic acid (aspirin) and salicylic acid induce multiple stress tolerance in bean and tomato plants. Plant Growth Regul.

[73] Clarke SM, Mur LAJ, Wood JE, Scott IM (2004). Salicylic acid dependent signaling promotes basal thermotolerance but is not essential for acquired thermotolerance in Arabidopsis thaliana. Plant J.

[74] Wang L-J, Li S-H (2006). Salicylic acid-induced heat or cold tolerance in relation to Ca2+ homeostasis and antioxidant systems in young grape plants. Plant Sci.

[75] Shahzad R, Khan AL, Bilal S, Waqas M, Kang S-M, Lee I-J (2017). Inoculation of abscisic acid-producing endophytic bacteria enhances salinity stress tolerance in Oryza sativa. Environ Exp Bot.

[76] Shahzad R, Waqas M, Khan AL, Asaf S, Khan MA, Kang S-M, Yun B-W, Lee I-J (2016). Seed-borne endophytic Bacillus amyloliquefaciens RWL-1 produces gibberellins and regulates endogenous phytohormones of Oryza sativa. Plant Physiol Biochem.

[77] Hildebrandt Tatjana M, Nunes Nesi A, Araújo Wagner L, Braun H-P (2015). Amino acid catabolism in plants. Mol Plant.

[78] Hildebrandt TM (2018). Synthesis versus degradation: directions of amino acid metabolism during Arabidopsis abiotic stress response. Plant Mol Biol.

[79] Barbosa MAM, AKdS L, DKY T, GDM V, KNN C, JRS B, MdCHdS M, RCLd C, BGd SF, CFd ON (2013). Bradyrhizobium improves nitrogen assimilation, osmotic adjustment and growth in contrasting cowpea cultivars under drought. Aust J Crop Sci.

[80] Vílchez JI, Niehaus K, Dowling DN, González-López J, Manzanera M (2018). Protection of Pepper Plants from Drought by Microbacterium sp. 3J1 by Modulation of the Plant's Glutamine and α-ketoglutarate Content: A Comparative Metabolomics Approach. Front Microbiol.

[81] Gagné-Bourque F, Bertrand A, Claessens A, Aliferis KA, Jabaji S (2016). Alleviation of Drought Stress and Metabolic Changes in Timothy (*Phleum pratense* L.) Colonized with Bacillus subtilis B26. Front Plant Sci.

[82] Santos AA, JAGd S, EdA G, Bonifacio A, Rodrigues AC, MdVB F (2018). Changes induced by co-inoculation in nitrogen–carbon metabolism in cowpea under salinity stress. Braz J Microbiol.

[83] Bhise KK, Bhagwat PK, Dandge PB (2017). Synergistic effect of Chryseobacterium gleum sp. SUK with ACC deaminase activity in alleviation of salt stress and plant growth promotion in *Triticum aestivum* L. 3 Biotech.

[84] de Ronde JA, Laurie RN, Caetano T, Greyling MM, Kerepesi I (2004). Comparative study between transgenic and non-transgenic soybean lines proved transgenic lines to be more drought tolerant. Euphytica.

[85] Georgieva K, Fedina I, Maslenkova L, Peeva V (2003). Response of <emph type="2">chlorina</emph> barley mutants to heat stress under low and high light. Funct Plant Biol.

[86] Lv W-T, Lin B, Zhang M, Hua X-J (2011). Proline accumulation is inhibitory to Arabidopsis seedlings during heat stress. Plant Physiol.

[87] Yuan L, Tang L, Zhu S, Hou J, Chen G, Liu F (2017). Influence of heat stress on leaf morphology and nitrogen--carbohydrate metabolisms in two wucai (Brassica campestris L.) genotypes. Acta Soc Bot Pol.

[88] Nakamoto H, Vigh L (2007). The small heat shock proteins and their clients. Cell Mol Life Sci.

[89] Hong S-W, Lee U, Vierling E (2003). Arabidopsis <em>hot</em> mutants define multiple functions required for acclimation to high temperatures. Plant Physiol.

[90] Chai C, Wang Y, Valliyodan B, Nguyen HT (2016). Comprehensive analysis of the soybean (Glycine max) GmLAX Auxin transporter gene family. Front Plant Sci.

[91] Deeken R, Geiger D, Fromm J, Koroleva O, Ache P, Langenfeld-Heyser R, Sauer N, May ST, Hedrich R (2002). Loss of the AKT2/3 potassium channel affects sugar loading into the phloem of Arabidopsis. Planta.

[92] Zhou L, He H, Liu R, Han Q, Shou H, Liu B (2014). Overexpression of GmAKT2 potassium channel enhances resistance to soybean mosaic virus. BMC Plant Biol.

[93] Khan MA, Asaf S, Khan AL, Jan R, Kang S-M, Kim K-M, Lee I-J (2020). Extending thermotolerance to tomato seedlings by inoculation with SA1 isolate of Bacillus cereus and comparison with exogenous humic acid application. PLoS One.

[94] Patten CL, Glick BR (2002). Role of <em>Pseudomonas putida</em> Indoleacetic acid in development of the host plant root system. Appl Environ Microbiol.

[95] Adhikari A, Lee KE, Khan MA, Kang SM, Adhikari B, Imran M, Jan R, Kim KM, Lee IJ (2020). Effect of silicate and phosphate solubilizing Rhizobacterium Enterobacter ludwigii GAK2 on Oryza sativa L. under cadmium stress. J Microbiol Biotechnol.

[96] Schwyn B, Neilands JB (1987). Universal chemical assay for the detection and determination of siderophores. Anal Biochem.

[97] Khan MA, Asaf S, Khan AL, Adhikari A, Jan R, Ali S, Imran M, Kim K-M, Lee I-J (2019). Halotolerant Rhizobacterial Strains Mitigate the Adverse Effects of NaCl Stress in Soybean Seedlings BioMed research international.

[98] Sambrook J, Russell D (2001). Molecular cloning: a laboratory manual.

[99] Tamura K, Stecher G, Peterson D, Filipski A, Kumar S (2013). MEGA6: molecular evolutionary genetics analysis version 6.0. Mol Biol Evol.

[100] Khan AL, Halo BA, Elyassi A, Ali S, Al-Hosni K, Hussain J, Al-Harrasi A, Lee I-J (2016). Indole acetic acid and ACC deaminase from endophytic bacteria improves the growth of Solanum lycopersicum. Electron J Biotechnol.

[101] Khan AL, Hamayun M, Kang S-M, Kim Y-H, Jung H-Y, Lee J-H, Lee I-J (2012). Endophytic fungal association via gibberellins and indole acetic acid can improve plant growth under abiotic stress: an example of Paecilomyces formosus LHL10. BMC Microbiol.

[102] Kang SM, Khan AL, Hamayun M, Shinwari ZKS, Kim YH, Joo GJ, Lee IJ (2012). *Acinetobacter calcoaceticus* ameliorated plant growth and influenced gibberellins and functional biochemicals. Pakistan J Botany.

[103] Vimala T, Poonghuzhali TV (2013). Estimation of pigments from seaweeds by using acetone and DMSO. Int J Sci Res.

[104] Genty B, Briantais J-M, Baker NR (1989). The relationship between the quantum yield of photosynthetic electron transport and quenching of chlorophyll fluorescence. Biochim Biophys Acta Gen Subj.

[105] Khan MA, Khan AL, Imran QM, Asaf S, Lee S-U, Yun B-W, Hamayun M, Kim T-H, Lee I-J (2019). Exogenous application of nitric oxide donors regulates short-term flooding stress in soybean. PeerJ.

[106] Seskar M, Shulaev V, Raskin I (1998). Endogenous methyl salicylate in pathogen-inoculated tobacco plants. Plant Physiol.

[107] Khan AL, Waqas M, Khan AR, Hussain J, Kang S-M, Gilani SA, Hamayun M, Shin J-H, Kamran M, Al-Harrasi A (2013). Fungal endophyte Penicillium janthinellum LK5 improves growth of ABA-deficient tomato under salinity. World J Microbiol Biotechnol.

[108] Kim Y, Mun B-G, Khan AL, Waqas M, Kim H-H, Shahzad R, Imran M, Yun B-W, Lee I-J (2018). Regulation of reactive oxygen and nitrogen species by salicylic acid in rice plants under salinity stress conditions. PLoS One.

[109] Marklund S, Marklund G (1974). Involvement of the superoxide anion radical in the autoxidation of pyrogallol and a convenient assay for superoxide dismutase. Eur J Biochem.

[110] Ellman GL (1959). Tissue sulfhydryl groups. Arch Biochem Biophys.

[111] Asaf S, Khan AL, Khan MA, Imran QM, Yun B-W, Lee I-J (2017). Osmoprotective functions conferred to soybean plants via inoculation with Sphingomonas sp. LK11 and exogenous trehalose. Microbiol Res.

[112] Chan C-X, Teo S-S, Ho C-L, Othman RY, Phang S-M (2004). Optimisation of RNA extraction from Gracilaria changii (Gracilariales, Rhodophyta). J Appl Phycol.

[113] Jan R, Khan MA, Asaf S, Lubna LI-J, Kim KM (2019). Metal resistant Endophytic Bacteria reduces cadmium, nickel toxicity, and enhances expression of metal stress related genes with improved growth of Oryza Sativa, via regulating its antioxidant machinery and endogenous hormones. Plants.

