# Supplementary figures and images for: Thermotolerance effect of plant growth-promoting Bacillus cereus SA1 on soybean during heat stress
Source: BMC Microbiol. 2020 Jun 22;20:175. doi: 10.1186/s12866-020-01822-7 (PMC7310250; doi:10.1186/s12866-020-01822-7)

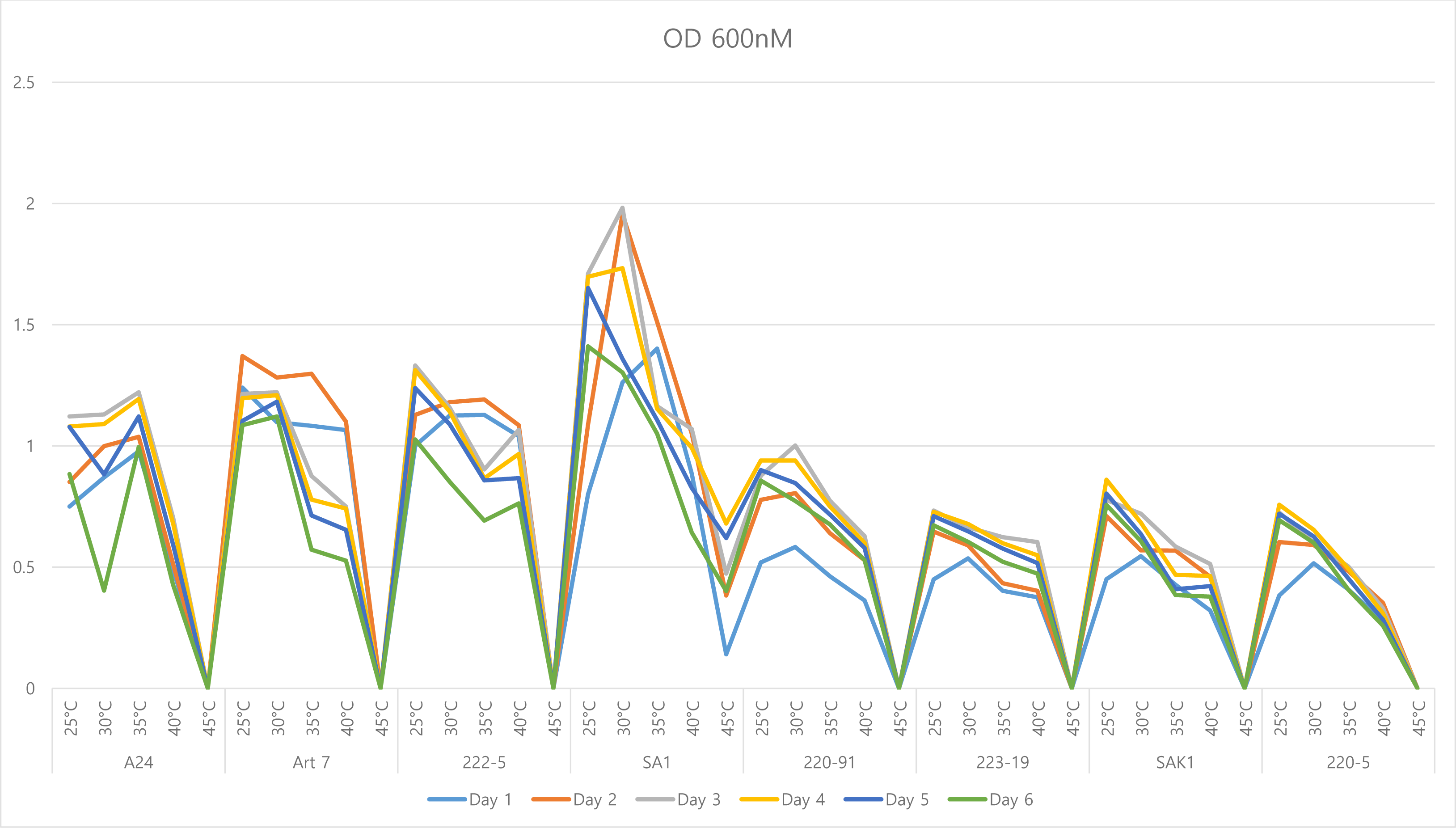

Supplement: Supplementary file 1 — Additional file 1: Figure S1. Growth of multiple plant growth-promoting traits producing endophytic bacteria (PGPEB). PGPEB were grown in LB media at 25 °C, 30 °C, 35 °C, 40 °C, and 45 °C for 6 days, and the growth was examined using a spectrophotometer at 600 nm. Each data point is the mean of three replicates. [file 12866_2020_1822_MOESM1_ESM.tif]

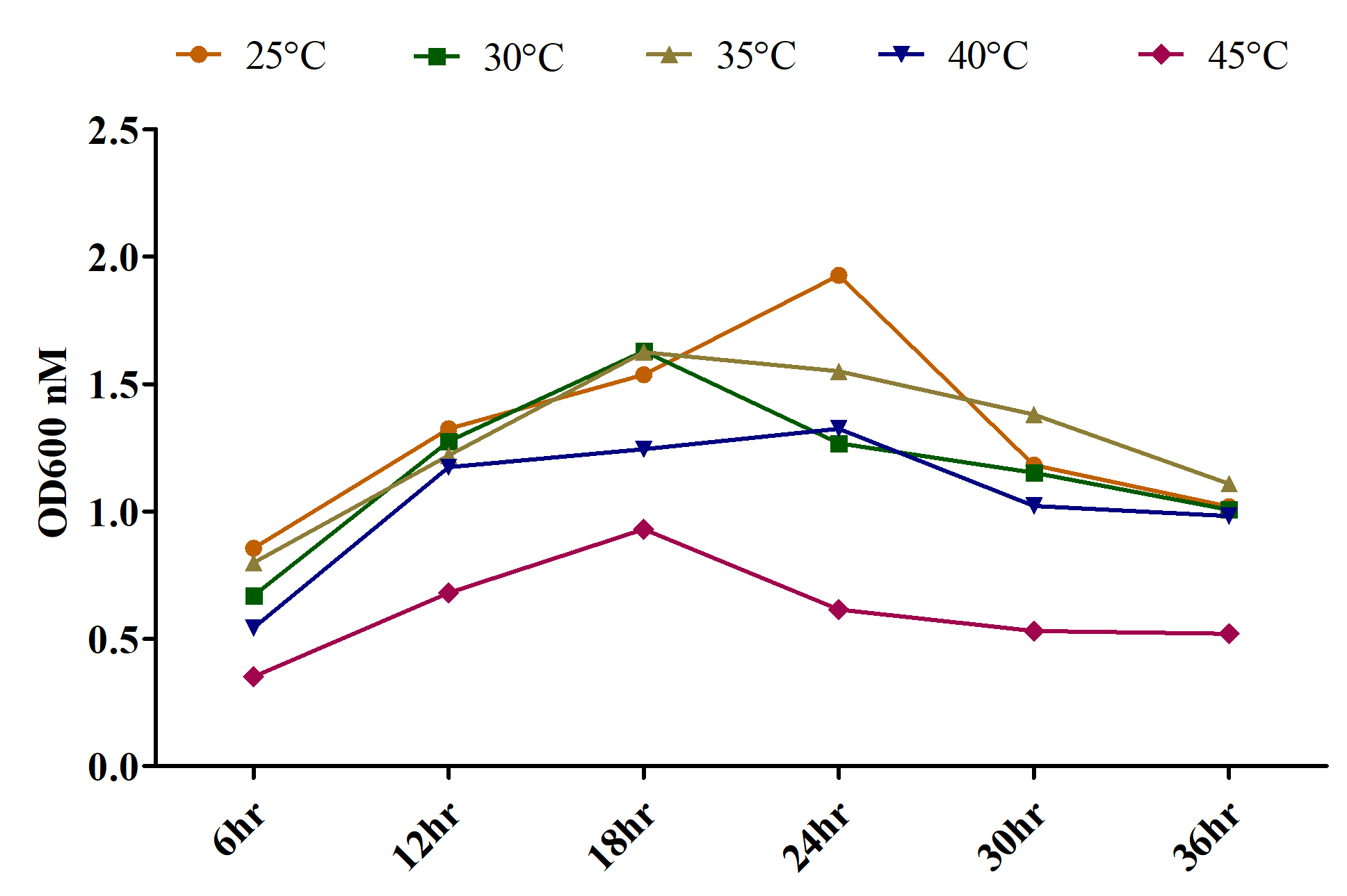

Supplement: Supplementary file 2 — Additional file 2: Figure S2. Growth of isolate SA1 in LB media. Isolate SA1 were grown in LB media at 25 °C, 30 °C, 35 °C, 40 °C, and 45 °C for 36 h, and the growth was examined using a spectrophotometer at 600 nm. Each data point is the mean of three replicates. [file 12866_2020_1822_MOESM2_ESM.jpg]
