# Supplementary material for: Thermotolerance effect of plant growth-promoting Bacillus cereus SA1 on soybean during heat stress
Source: BMC Microbiol. 2020 Jun 22;20:175. doi: 10.1186/s12866-020-01822-7 (PMC7310250; doi:10.1186/s12866-020-01822-7)
Supplement: Supplementary file 3 — Additional file 3: Table S1. Description of plants species and number of their yielded endophytic isolates. The isolates were preliminary sorted for single or multiple plant growth beneficial activities. [file 12866_2020_1822_MOESM3_ESM.docx]

| **Plants Name** | **No of isolates** | **Isolates having single Plant Growth Promoting characteristics** | | | **Isolates with multiple PGP characteristics** |
| --- | --- | --- | --- | --- | --- |
|  |  | **IAA production** | **Siderophore** | **Phosphate** |  |
| **Endophytes isolates** | | | | | |
| *Artemisia princeps* Pamp. | 24 | 16 | 5 | 6 | 8 |
| *Chenopodium ficifolium* Smith. | 6 | 1 | 0 | 2 | 0 |
| *Oenothera biennis* L. | 17 | 12 | 1 | 2 | 1 |
| *Echinochloa crus-galli* (L.) Beauv. | 12 | 7 | 1 | 3 | 4 |

**S. Table 1.** Description of plants species and number of their yielded endophytic isolates. The isolates were preliminary sorted for single or multiple plant growth beneficial activities.
