# Supplementary material for: Thermotolerance effect of plant growth-promoting Bacillus cereus SA1 on soybean during heat stress
Source: BMC Microbiol. 2020 Jun 22;20:175. doi: 10.1186/s12866-020-01822-7 (PMC7310250; doi:10.1186/s12866-020-01822-7)
Supplement: Supplementary file 4 — Additional file 4: Table S2. List of primers used in this study [file 12866_2020_1822_MOESM4_ESM.docx]

**S. Table 2.** List of primers used in this study

| **Primers** | | |
| --- | --- | --- |
| ***GmHSP*** | F | 5′ATGTCTCTGATTCCAAGTATTTTCGGT3′ |
|  | R | 5′TTAACCAGAGATTTCAATGGCCTTAAC3′ |
| ***GmLAX3*** | F | 5′CTGGCAGGGTTTTGCATTAT3′ |
|  | R | 5′GCCTGTGCATTTCATAGCAA3′ |
| ***GmAKT2*** | F | 5′CACCATGCTAGCTGATCGTTACCC3′ |
|  | R | 5′TCAGCTTATCCAACAAAAAAAAAGT3′ |
| ***Actin*** | F | 5′ATGGTGGGTATGGGTCAAAA3′ |
|  | R | 5′GAGGACAGGATGCTCCTCAG3′ |
